# The quinoxaline di-N-oxide DCQ blocks breast cancer metastasis *in vitro* and *in vivo* by targeting the hypoxia inducible factor-1 pathway

**DOI:** 10.1186/1476-4598-13-12

**Published:** 2014-01-24

**Authors:** Khaled Ghattass, Sally El-Sitt, Kazem Zibara, Saide Rayes, Makhluf J Haddadin, Marwan El-Sabban, Hala Gali-Muhtasib

**Affiliations:** 1 Department of Biology, Faculty of Arts and Sciences, American University of Beirut, Beirut, Lebanon; 2 Department of Biology, Doctoral School of Science and Technology, Lebanese University, Beirut, Lebanon; 3 Department of Chemistry, Faculty of Arts and Sciences, American University of Beirut, Beirut, Lebanon; 4 Department of Anatomy, Cell Biology and Physiological Sciences, Faculty of Medicine, American University of Beirut, Beirut, Lebanon

**Keywords:** Breast cancer, Hypoxia, Metastasis, HIF-1α, DCQ

## Abstract

**Background:**

Although tumor hypoxia poses challenges against conventional cancer treatments, it provides a therapeutic target for hypoxia-activated drugs. Here, we studied the effect of the hypoxia-activated synthetic quinoxaline di-N-oxide DCQ against breast cancer metastasis and identified the underlying mechanisms.

**Methods:**

The human breast cancer cell lines MCF-7 (p53 wildtype) and MDA-MB-231 (p53 mutant) were treated with DCQ under normoxia or hypoxia. Drug toxicity on non-cancerous MCF-10A breast cells was also determined. *In vitro* cellular responses were investigated by flow cytometry, transfection, western blotting, ELISA and migration assays. The anti-metastatic effect of DCQ was validated in the MDA-MB-231 xenograft mouse model.

**Results:**

DCQ selectively induced apoptosis in both human breast cancer cells preferentially under hypoxia without affecting the viability of non-cancerous MCF-10A. Cancer cell death was associated with an increase in reactive oxygen species (ROS) independently of p53 and was inhibited by antioxidants. DCQ-induced ROS was associated with DNA damage, the downregulation of hypoxia inducible factor-1 alpha (HIF-1α), and inhibition of vascular endothelial growth factor (VEGF) secretion. In MCF-7, HIF-1α inhibition was partially *via* p53-activation and was accompanied by a decrease in p-mTOR protein, suggesting interference with HIF-1α translation. In MDA-MB-231, DCQ reduced HIF-1α through proteasomal-dependent degradation mechanisms. HIF-1α inhibition by DCQ blocked VEGF secretion and invasion in MCF-7 and led to the inhibition of TWIST in MDA-MB-231. Consistently, DCQ exhibited robust antitumor activity in MDA-MB-231 breast cancer mouse xenografts, enhanced animal survival, and reduced metastatic dissemination to lungs and liver.

**Conclusion:**

DCQ is the first hypoxia-activated drug showing anti-metastatic effects against breast cancer, suggesting its potential use for breast cancer therapy.

## Background

Inadequate oxygen delivery to rapidly growing cancers forms heterogeneously distributed hypoxic regions within solid tumors [1,2]. The severity of oxygen deficiency in such regions is an independent marker of poor prognosis and is correlated clinically and experimentally with the resistance of tumors to chemo- and radio-therapies [3]. Additionally, intra-tumoral hypoxia contributes positively to sequential steps of metastasis and aids in the acquisition and maintenance of cancer stem cells (CSC) [3-7]. The master regulator of transcriptional responses to hypoxic stress is the hetero-dimeric hypoxia induced transcription factor (HIF). HIF is composed of two subunits, an alpha subunit (HIF-1α) and a beta subunit (HIF-1β). HIF-1β subunit is constitutively expressed, while the control of HIF response is achieved primarily *via* proteasomal-dependent degradation of the α subunit [8]. Initially, the degradation was thought to occur only in an oxygen-dependent manner; however, several oxygen-independent mechanisms have been described [9,10]. Increased levels of HIF-1α are associated with increased refractiveness of several solid tumors to conventional therapies [11].

Transcriptional targets of HIF-1α include major regulators of key processes including angiogenesis, epithelial to mesenchymal transition (EMT), which together lead to metastasis [3,4,11,12]. More recently, HIF-1α was shown to enhance signaling pathways activated in CSCs, favoring their enrichment within solid tumors [13,14]. Because hypoxic responses in cancer cells are primarily mediated by hypoxia inducible factors, targeting HIF-1α directly or indirectly or eradicating intra-tumoral hypoxic regions are viable strategies to inhibit aggressive tumors [8,11,15]. Despite such significant challenges posed by tumor hypoxia, the reductive nature of the hypoxic microenvironment was exploited for selective activation of several drug classes including aromatic N-oxides [11,16]. These drugs undergo reduction to produce a transient radical intermediate, which, in the presence of oxygen, is back oxidized to the non-toxic pro-drug, hence minimizing side effects to normal non-hypoxic tissues [16]. The most studied hypoxia-activated drug is tirapazamine (TPZ). TPZ has reached clinical trials in combination with other drugs against several cancers; however, it shows moderate activity against breast cancer, which is known to bear severely hypoxic regions [17].

We have identified a potent compound that shares the di-N-oxide moiety with TPZ. This molecule, 2-benzoyl-3-phenyl-6,7-dichloroquinoxaline 1,4-dioxide (DCQ) inhibits the viability of several cancer cell lines with a greater efficacy under hypoxia [18-24]. Additionally, we have previously shown that DCQ reduces HIF-1α and hypoxia-induced angiogenesis; however, the mechanism by which DCQ exerts its effect is still unknown [23]. Hypoxia-activated drugs can target solid tumors by either killing resistant cells residing in the hypoxic niche or by modulating hypoxia-induced pathways involved in cancer progression. Here, we investigated the anti-metastatic activity of DCQ against breast cancer using two human breast cancer cell lines that differ in their p53 status, and identified the underlying mechanisms involved. We show that the antitumor activity and pro-apoptotic effects of DCQ are selective to cancer cells and involve the generation of ROS and suppression of HIF-1α protein expression *via* different mechanisms in the two cell lines. The ability of DCQ to inhibit breast cancer metastasis and HIF-1α expression was validated in the xenograft model of sub-dermally injected MDA-MB-231 cells in immune-compromised NOD/SCID mice.

## Results and discussion

### Results

#### DCQ selectively reduces cell viability and inhibits colony formation mostly under hypoxia

**Figure 1:**
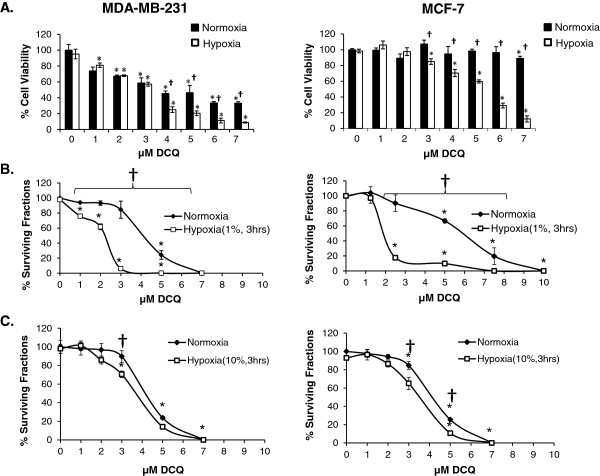
**DCQ reduces the viability and colony forming ability of breast cancer cell lines (MDA-MB-231 and MCF-7). (A)** MTT viability assay was performed after 6 hours of exposure to DCQ under normoxia (21%) or hypoxia (1%). Results (Average ± SE) are from triplicate measurements from 3 independent experiments. One-way ANOVA was used to compare DCQ-treated *versus* control and statistical significance of p < 0.05 is indicated by *. Two sample t-test was used to compare between the effect of DCQ under normoxia *versus* hypoxia under the same concentration, p < 0.05 is indicated by †. **(B,C)** Clonogenic survival assay of cells exposed to different concentrations of DCQ under normoxia or hypoxia. Cells were treated with DCQ and incubated for 3 hours after which they were trypsinized and re-plated at a density of 300 cells/100 mm petri dish and surviving colonies (>50 cells) were counted 12 days after plating. *p < 0.05 significance with respect to control (one way ANOVA). †p < 0.05 significant difference between normoxia and hypoxia at the same DCQ concentration (two sample t-test). Results (Average ± SE) are from duplicate measurements of 3 independent experiments.

Previous studies in our laboratory showed that DCQ is a potent hypoxic cytotoxin and pro-apoptotic drug in several murine and human cancer cell lines [18-24]. However, its anti-metastatic potential has not been investigated against human breast cancer, which is known to harbor severely hypoxic regions [25,26]. MTT assay showed that DCQ reduced the viability of human breast cancer MCF-7 and MDA-MB-231 cell lines in a dose- and time-dependent manner (Figure 1 and data not shown). The p53 wild type MCF-7 cell line was more resistant (hypoxia IC_50_ = 5 μM *vs* 3 μM in MDA-MB-231) and hypoxia enhanced drug efficacy in this cell line, while the aggressive MDA-MB-231 was sensitive under both conditions at 6 hours of exposure (Figure 1A). Interestingly, DCQ was not toxic to non-cancerous MCF-10A [27] under normoxic conditions (Additional file 1: Figure S1).

Clonogenic survival assay (Figure 1B) confirmed the oxygen-dependent cytotoxic activity of DCQ. Treatment with DCQ (1–10 μM) reduced colony formation more significantly under hypoxia than normoxia (Figure 1B). Increasing the oxygen levels to 10% suppressed the hypoxia-selectivity of DCQ (Figure 1C). Corresponding to 1% and 10% oxygen, the hypoxia cytotoxicity ratio (HCR) was 3.4 and 1.25 in MCF-7, and 2 and 0.9 in MDA-MB-231, respectively (Figure 1B and 1C). In accordance with the cell viability results, clonogenic survival showed that the invasive MDA-MB-231 was more sensitive, whereby 4 μM DCQ induced 100% inhibition in colony formation, however, in MCF-7 this was achieved at 7.5 μM DCQ (Figure 1B).

To compare DCQ and TPZ activity against human breast cancer cell lines, we investigated drug effects on MCF-7 and MDA-MB-231 using trypan blue and MTT assays. In line with the obtained results, 5 μM DCQ under hypoxia significantly reduced the viability of MCF-7 and MDA-MB-231 (Additional file 1: Figure S2); however, the cytotoxic activity of TPZ did not reach statistical significance even at relatively high concentrations (20 μM) under both normoxic and hypoxic conditions (Additional file 1: Figure S2). This led us to drop further comparative experiments between DCQ and TPZ.

#### DCQ induces apoptosis and DNA damage more significantly under hypoxia

**Figure 2:**
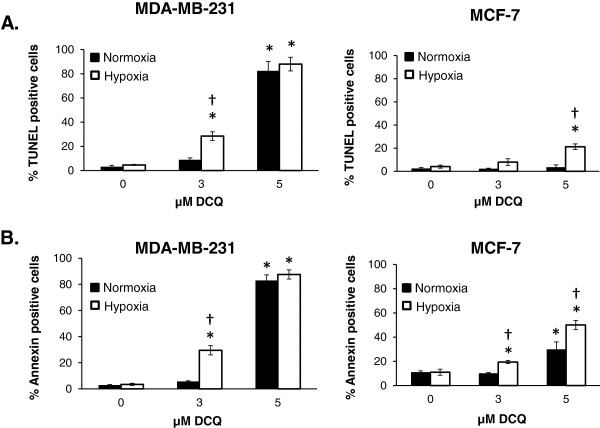
**Differential sensitivity of MDA-MB-231 and MCF-7 to DCQ-induced apoptosis. (A)** Cells were treated with DCQ for 6 hours under normoxia or hypoxia. After 24 hours, the extent of DNA fragmentation was determined by TUNEL assay and measured by flow cytometry. **(B)** Cells were treated with DCQ for 6 hours under normoxia or hypoxia. After 24 hours control and DCQ treated cells were stained with fluorescein-conjugated annexin V and PI; apoptotic and necrotic cells were analyzed by flow cytometry. The percentage of apoptotic cells was determined in response to DCQ using CellQuest software and the averages ± SE were obtained from the results of three independent experiments each done in duplicate. Two sample t-test was used to compare between the effect of DCQ under normoxia *versus* hypoxia under the same concentration, p < 0.05 is indicated by †. The effect of the same concentration of DCQ (3 μM and 5 μM) on the two cell lines was compared by t-test. MCF-7 cells were more resistant to DCQ apoptotic effect than the MDA-MB-231 (p < 0.05).

We then used IC_50_ concentrations of DCQ (3 μM in MDA-MB-231; 5 μM in MCF-7) to investigate drug effects on cell cycle by flow cytometric analysis of fluorescent PI-stained cellular DNA (Additional file 1: Figure S3). Cells exposed to DCQ for 24 hours under normoxia or hypoxia (6 hours in 1% oxygen followed by 18 hours in normoxia) exhibited an increase in the pre G_1_ population more significantly under hypoxia than normoxia (Additional file 1: Figure S3). Since the accumulation of cells in pre G_1_ may be due to apoptosis, TUNEL and annexin V/PI assays were performed to confirm the mode of cell death. In both cell lines, the extent of DCQ-induced apoptosis was greater under hypoxia than normoxia (28% *vs* 9% in MDA-MB-231; 21% *vs* 3% in MCF-7) (Figure 2A). This enhanced apoptosis under hypoxia was confirmed by Annexin V/PI assay (Figure 2B), suggesting that the increases in pre G_1_ observed by flow cytometry are due to apoptosis. Higher DCQ concentrations of 5 μM caused more than 80% apoptosis in MDA-MB-231 and loss of difference in drug efficacy between normoxia and hypoxia. Consistent with viability and colony formation assay, the p53 wild type MCF-7 cells were more resistant to the pro-apoptotic effects of DCQ (p < 0.05, Figure 2).

**Figure 3:**
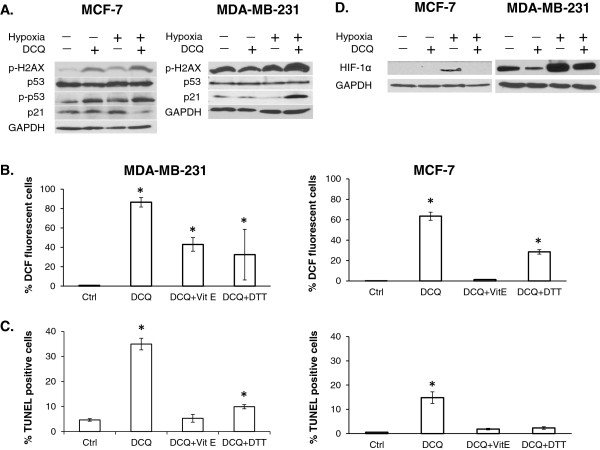
**DCQ induces DNA damage and apoptosis via reactive oxygen species. (A)** Whole cell lysates of MCF-7 and MDA-MB-231 were prepared after 6 hours of exposure to DCQ (5 μM) under normoxia or hypoxia, and blots were probed for p-H2AX, p53, p-p53, p21, HIF-1α and GAPDH. Results are representative of three independent experiments. **(B)** MDA-MB-231 and MCF-7 cells were pretreated with 1 mM Vitamin E or DTT for 2 hours followed by 25 min incubation with 10 μM CM-H_2_DCFDA dye. Cells were washed with PBS and treated with DCQ for 1 hour under normoxia or hypoxia, after which cells were harvested and the amount of DCF fluorescence was analyzed by flow cytometry. Each percentage is the average ± SE of three independent experiments. **(C)** MDA-MB-231 and MCF-7 cells were pretreated with 1 mM Vitamin E or DTT for 2 hours followed by 6 hours treatment with DCQ under normoxia or hypoxia. After 24 hours, the extent of DNA fragmentation was determined by TUNEL assay and measured by flow cytometry. One-way ANOVA was used to compare DCQ-treated *versus* control and statistical significance of p < 0.05 is indicated by *. **(D)** Whole cell lysates of MCF-7 and MDA-MB-231 were prepared after 6 hours of exposure to DCQ (5 μM) under normoxia or hypoxia, and blots were probed for HIF-1α and GAPDH. Results are representative of three independent experiments.

We have shown that DCQ induces DNA damage and the DNA damage response signaling pathway leading to apoptosis [19,24]. To confirm that DCQ exerts its antitumor effect in MCF-7 and MDA-231 by a similar mechanism, we investigated changes in the levels of gamma-H2AX, a marker of DNA damage [28]. Under hypoxic conditions, 5 μM DCQ elicited a marked increase in gamma-H2AX protein in both cell lines (Figure 3A). We then investigated whether DCQ-induced DNA damage activates p53 in MCF-7 cells, as in human colon cancer cells [19]. No changes in total p53 protein were observed; however, in accordance with gamma-H2AX results, the phosphorylation levels of p53 (also a marker of DNA damage) were increased by DCQ, especially under hypoxia. Considering that MDA-MB-231 cells harbor a p53 dominant-negative mutation, there was no distinct band of p-p53 and no changes in p53 protein expression were induced by DCQ in this cell line (Figure 3A).

#### DCQ induces apoptosis *via* the generation of reactive oxygen species

Quinoxaline dioxides are known to cause DNA damage either *via* direct interaction with DNA or through the induction of ROS [11,16,17]. To investigate whether DNA damage by DCQ is associated with an increase in intracellular ROS, we measured ROS by the dichlorofluorescin assay in MCF-7 and MDA-MB-231 cells exposed to DCQ under hypoxia (Figure 3B). An oxidant shift was observed in MCF-7 and MDA-MB-231 cells after 1 hour of treatment with DCQ, and the extent of ROS production was greater in MDA-MB-231 (95%) in comparison to MCF-7 (60%) cells, which may explain the enhanced drug sensitivity of MDA-MB-231. A 2 hour pretreatment with the strong antioxidant vitamin E or the reducing agent DTT suppressed DCQ-induced ROS levels by 40-100% in both cell lines (Figure 3B). In addition, both antioxidants significantly protected breast cancer cells against DCQ-induced apoptosis (Figure 3C). The percentage of TUNEL positive cells decreased to control levels upon pretreatment with vitamin E or DTT (Figure 3C), suggesting that intracellular ROS play a role in DCQ-induced apoptosis. HPLC and thin layer chromatography showed no direct drug interaction between the antioxidants and DCQ (data not shown).

#### DCQ antitumor activity in MCF-7 and MDA-MB-231 is through inhibition of HIF-1α *via* different mechanisms

We have shown that the hypoxia-induced cytotoxic activity of DCQ is associated with the reduction of HIF-1α accumulation [18,19,23,24]. However, the mechanism by which DCQ suppresses HIF-1α is poorly understood. In p53 wild type MCF-7 cells, HIF-1α band was only detected under hypoxia and its expression was abrogated in response to DCQ (Figure 3D). In MDA-MB-231 cells; however, HIF-1α was constitutively expressed under normoxia, and its expression was markedly increased under hypoxia. The increase in HIF-1α protein in MDA-MB-231 was reduced by DCQ (Figure 3D). The effect of ROS on HIF-1α was studied by pretreating MDA-MB-231 cells with DTT followed by DCQ under hypoxia. Pretreatment with the reducing agent DTT did not reverse the inhibitory effect of DCQ on HIF-1α protein, suggesting a ROS independent mechanism of HIF-1α regulation (Additional file 1: Figure S4).

**Figure 4:**
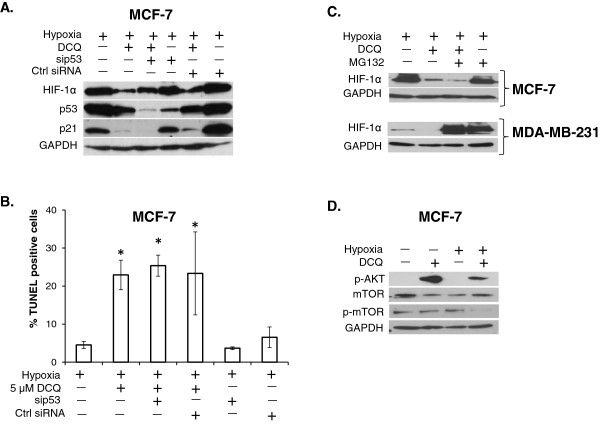
**DCQ reduces HIF-1α through different mechanisms in MCF-7 and MDA-MB-231. (A)** MCF-7 cells were transfected with siRNA against p53 and ctrl siRNA (scrambled sequence) using lipofectamine 2000. After 24 hours, cells were treated with DCQ (5 μM) for 6 hours under hypoxia. Whole cell lysates were prepared, and blots were probed against indicated antibodies. **(B)** MCF-7 cells were transfected with siRNA against p53 and ctrl siRNA (scrambled sequence) using lipofectamine 2000. Transfected cells were treated with DCQ (5 μM) for 6 hours under hypoxia. The extent of DNA fragmentation was determined by TUNEL assay 24 hours later using flow cytometry**.** One-way ANOVA was used to compare DCQ-treated *versus* control and statistical significance of p < 0.05 is indicated by *. **(C)** Whole cell lysates were prepared after pretreating MCF-7 and MDA-MB-231 with the proteasome inhibitor MG132 (3 μM) then treated with DCQ, and blots were probed for HIF-1α. Results are from three independent experiments. **(D)** Whole cell lysates of MCF-7 were prepared after 6 hours of exposure to DCQ (5 μM) under normoxia or hypoxia, and blots were probed for p-AKT, mTOR, p-mTOR and GAPDH.

It was shown that in MCF-7 cells, DNA damaging agents activate p53, which in turn targets HIF-1α to proteasomal-dependent degradation [28]. Having shown that DCQ induces DNA damage in this cell line, we assessed whether p53 plays a similar role in DCQ-induced downregulation of HIF-1α. We hypothesized that, if the activation of p53 by DCQ targets HIF-1α to degradation, then silencing p53 would reverse this effect and protect against HIF-1α down regulation. To address this, MCF-7 cells were transfected with siRNA against p53 and the levels of HIF-1α and p21 were assessed by western blot (Figure 4A). Under hypoxia, DCQ caused a reduction in HIF-1α protein in non-transfected and control MCF-7 cells. However, silencing p53 by siRNA did not completely reverse the effect of DCQ on HIF-1α, suggesting that p53 is not the major mediator of DCQ-induced downregulation of HIF-1α (Figure 4A). DCQ-induced down regulation of p21 was also not affected by p53 silencing, indicating a p53-independent mechanism for the decrease of p21 in MCF-7 cells (Figure 4A).

Additionally, we investigated the role of p53 in DCQ-induced apoptosis. Interestingly, DCQ increased the percentage of TUNEL positive cells under hypoxia from 5 to 23%, and this was not reduced upon silencing of p53 (25%), confirming that DCQ induces apoptosis in a p53-independent manner (Figure 4B). We investigated whether DCQ is targeting HIF-1α to proteasomal-dependent degradation by pretreating MCF-7 and MDA-MB-231 with the proteasome inhibitor MG132. As expected, in MCF-7, DCQ reduced the levels of HIF-1α in an MG132-independent manner (in line with p53 independent role), indicating a proteasomal-independent down regulation of HIF-1α (Figure 4C). However, in MDA-MB-231, DCQ reduced HIF-1 α to control levels, and this was abrogated by pretreatment of cells with MG132, suggesting that the proteasome is involved in DCQ-induced down regulation of HIF-1α in this cell line (Figure 4C).

Since in MCF-7 cells the proteasome was not involved in DCQ-induced HIF-1α inhibition, we investigated whether other upstream regulators of HIF-1α translation are responsible for this inhibition. It is known that the activation of PI3-AKT-mTOR pathway shifts the balance towards the accumulation of HIF-1α *via* increased translational activity [3]. Moreover, recent reports have shown that TPZ down regulates HIF-1α by inhibiting its translation through enhanced phosphorylation of AKT and reduction of mTOR phosphorylation [29]. In MCF-7 cells, we show that DCQ increases the phosphorylation of AKT, and reduces the levels of phosphorylated mTOR, suggesting that the downregulation of HIF-1α in this cell line is *via* decreased synthesis rather than activation of proteasomal degradation (Figure 4D).

#### DCQ significantly reduces hypoxia-induced TWIST, VEGF secretion and migration

**Figure 5:**
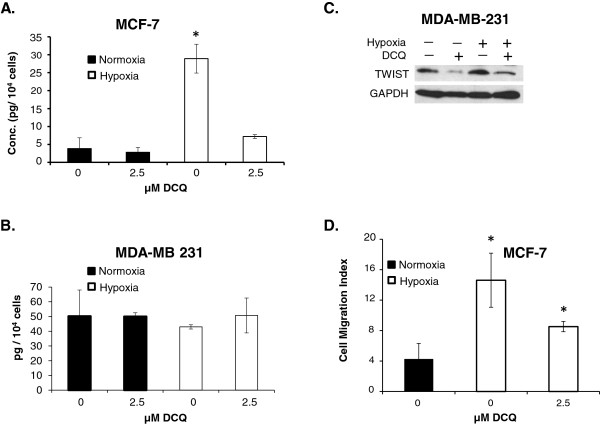
**DCQ reduced HIF-1α downstream targets. (A, B)** ELISA for secreted VEGF in MCF-7 cells and MDA-MB-231 after 12 hours of exposure to DCQ under normoxia or hypoxia. Since 6 hours were insufficient to induce a difference in the secreted VEGF upon exposure to hypoxia, we treated with a lower concentration of DCQ for 12 hours. Results (Average ± SE) are of duplicate measurements of two independent experiments. One-way ANOVA was used to compare DCQ-treated *versus* control and statistical significance of p < 0.05 is indicated by *. **(C)** MDA-MB-231 cells were treated with DCQ (5 μM) for 6 hours either under normoxia or under hypoxia. Whole cell lysates were prepared, and blots were probed against TWIST. **(D)** Trans-well migration assay for cells after 24 hours of exposure to DCQ under normoxia and hypoxia. 2 x 10^4^ MCF-7 cells were seeded on top of matrigel apical insert in serum free media and the lower chamber (the will) was filled with 10% FBS media. Cells were either treated with 2.5 μM DCQ or vehicle (supplemented in the upper chamber only) under normoxia and hypoxia for 24 hours. Then the lower part of the gel was fixed in 4% formaldehyde and cells were stained with Hoechst and counted using fluorescence microscopy. The averages ± SE were obtained from the results of two independent experiments. One-way ANOVA was used to compare DCQ-treated *versus* control and statistical significance of p < 0.05 is indicated by *.

Hypoxia-induced increase in VEGF expression is controlled by HIF-1α [3,10]. The levels of secreted VEGF were examined by ELISA and were shown to be significantly higher in supernatants of MCF-7 after exposure to hypoxia for 12 hours, an effect that was completely reversed by treating the cells with 2.5 μM DCQ (Figure 5A,B). DCQ had no significant effect on VEGF secretion in MDA-MB-231, possibly due to the fact that VEGF secretion is hypoxia-independent in this cell line (Figure 5A,B).

Another target gene for HIF-1α involved in EMT is TWIST. MDA-MBA-231 exhibit hypoxia-independent elevated levels of TWIST, which is attributed to the high basal levels of HIF-1α even under normoxia and is thought to be responsible for the high invasive potential of this cell line (Figure 3D) [12]. In line with the DCQ-induced reduction in HIF-1α in MDA-MB-231, TWIST levels were down regulated upon DCQ treatment under normoxia and hypoxia (Figure 5B). Under the same conditions, TWIST was undetectable by western blot in MCF-7 cells.

Hypoxia enhances cell motility *via* several pathways including HIF-1α [3]. To investigate whether the reduction in HIF-1α by DCQ translates into inhibition in migration, we conducted trans-well migration assay by seeding MCF-7 and MDA-MB-231 cells over serum-reduced matrigel for 24 hours under normoxia and hypoxia. Incubation of cells under hypoxia for 6 hours was insufficient to induce a change in the motility of MCF-7 cells, however, 24 hours exposure to hypoxia led to a 4-fold increase in the relative number of migrating MCF-7 (Figure 5D) but not MDA-MB-231 (data not shown), due to the hypoxia-independent migration ability of the latter cell line [27]. Since 5 μM DCQ for 24 hours induced significant cell death in MCF-7 cells, we studied the motility of this cell line in response to 2.5 μM DCQ and showed significant reduction in hypoxia-induced cell motility (Figure 5D).

#### DCQ exhibits anti-metastatic activity *in vivo*

**Figure 6:**
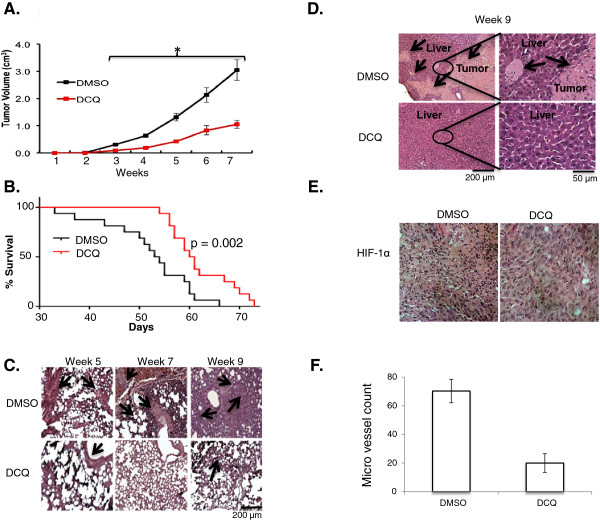
**DCQ reduced tumor volume and metastasis and increases survival *in vivo*.***In vivo* model used to test the effect of DCQ, briefly 4 x 10^6^ MDA-MB-231 were sub-dermally injected under the dorsal neck region of NOD-SCID mice. One week later mice were pooled into two groups DCQ treated and control. **(A)** Effect of DCQ on tumor growth of sub-dermally injected MDA-MB-231 mice. The growth of the primary tumor was monitored by measuring its dimensions on a weekly basis. Tumor volume (V, cm^3^) was determined by the equation: V = length × width × height). Data was plotted as the average tumor size of 2 separate experiments (10 mice/group/experiment) and error bars represent the standard error of the means. One-way ANOVA was used to compare DCQ-treated *versus* control and statistical significance of p < 0.05 is indicated by *. **(B)** Effect of DCQ on the survival of MDA-MB-231 injected mice. The survival curves of sub-dermally injected NOD-SCID mice by MDA-MB-231 cells, with DCQ treatment (17 mg/Kg in 50 μL DMSO) or control (in 50 μL DMSO). Experiment performed once with 22 mice per treatment group, significant difference was tested by Log-Rank Mantel-Cox test, p value = 0.002. **(C)** H&E staining of the lung of s.d. injected MDA-MB-231 mice was performed. Arrows indicate infiltration. **(D)** H&E staining of the liver samples of *s.d.* injected MDA-MB-231 mice was performed. Arrows indicate infiltration. **(E)** Photograph of primary tumor from control and DCQ-treated mice with immunohistochemistry staining against HIF-1α after 5 weeks. **(F)** Micro vessel density of cross section of primary tumors from control and DCQ-treated mice after 5 weeks. Results are from two mice per treatment group. Average ± SE are plotted form blinded count low magnification.

We validated the effect of DCQ *in vivo* using our established experimental metastasis mouse model. Briefly, 4x10^6^ MDA-MB-231 cells were injected *s.d.* into the subcutaneous area of the neck region of NOD-SCID mice. DCQ (17 mg/Kg in 50 μL DMSO) or DMSO (50 μL as control) were administered *i.p.*, twice per week for 4 weeks, and tumor volume was monitored weekly. Starting 3 weeks post treatment, tumor growth was significantly reduced by DCQ (p < 0.0001) (Figure 6A). Moreover, DCQ-treated mice showed significant improvement in their overall survival (p < 0.01). Indeed, around 50% of DCQ-treated mice survived beyond 60 days, time at which most DMSO-treated mice had already died (Figure 6B).

We further studied if the effect of DCQ on survival and tumor volume correlated with reduced metastasis to the lungs and liver. Metastasis from the site of injection (primary site) to the lungs and liver was studied by histology at 5, 7 or 9 weeks post tumor cell injection (Figure 6C). The infiltration of tumor cells was observed in the lungs of untreated mice at 5 weeks, which increased considerably at 7 weeks and reached its highest level at 9 weeks (Figure 6C). On the other hand, no or less infiltration was observed in the lungs of DCQ-treated mice (Figure 6C). The liver, another common secondary site of breast cancer metastasis, was also examined for tumor infiltration. Liver metastases were detected in DMSO-treated mice after 9 weeks; however, no liver metastasis was observed in DCQ-treated mice (Figure 6D). A marked difference in the lungs and livers of drug treated mice compared to control mice was observed at all time intervals (Figure 6C, D). Additionally, immunohistochemistry of primary tumor tissue showed that DCQ blocked the accumulation of HIF-1α (Figure 6E), and significantly reduced the micro vessel density within the primary tumor, as measured by anti-PECAM antibody (Figure 6F). These data suggest that DCQ suppresses metastasis *in vivo* and confirm the previously reported anti-angiogenic activity of DCQ against mouse breast tumors *in vivo*[23].

### Discussion

Breast cancer is the most frequently diagnosed malignancy in women. Heterogeneous distribution of hypoxic regions is commonly detected within invasive breast tumors [14,26]. These resistant regions are known to have larger tumor sizes, and to consist of worse grade tumors that are invasive and metastatic [25]. The use of hypoxia-activated drugs is among the leading approaches to circumvent hypoxia challenges [16]. This strategy provides selective tumor suppression, thus offering minimal toxicity to non-hypoxic tissues. Several hypoxia-activated compounds have reached clinical trials for the treatment of a variety of solid tumors [30-36]. However, so far these drugs have shown moderate to low activity against breast cancer. Here, we document for the first time that DCQ is a hypoxia-activated cytotoxin effective against breast cancers that harbor different p53 status and have different invasive potential. We show that DCQ induces apoptosis and reduces HIF-1α regulated pathways. In human breast cancer xenograft model, DCQ attenuated metastasis to the lungs and liver and significantly reduced tumor growth as compared to control mice (DMSO-treated). Additionally, DCQ prolonged the survival of 50% of treated mice beyond 60 days, by that time, however, 90% of untreated mice have already died. These data suggest that targeting breast cancer with the hypoxia-activated drug DCQ is a potential therapeutic approach to suppress metastasis through the reduction of HIF-1α and downstream activated pathways.

It has been shown that DCQ exerts its cytotoxic activity through different cellular mechanisms in a cell-type specific manner [18-22]. In this study, DCQ was found to reduce the viability and colony forming ability of MCF-7 and MDA-MB-231 *via* the induction of apoptosis, an effect that was significantly enhanced under hypoxia. Interestingly, the p53 mutant and invasive MDA-MB-231 cell line was more sensitive to DCQ than p53 wild type MCF-7 cells. In order to rule out the possible involvement of p53 in drug sensitivity, we silenced functional p53 in MCF-7 cells, and found that DCQ-induced apoptosis was not enhanced, suggesting that the higher drug sensitivity in MDA-MB-231, which provides additional selectivity for tumor versus normal tissue toxicity, may not be attributed to p53 status. This extends previous observations that DCQ-induced apoptosis in HCT116 human colon cancer cells is independent of p53 [19], rendering DCQ a promising molecule, especially when considering that more than 50% of human cancers bear mutated or abnormal p53.

Most hypoxia-activated drugs undergo bio-reductive activation, which generates intra cellular reactive radicals, namely hydroxyl radicals that break cellular DNA ultimately leading to apoptosis [16,17]. Using alkaline comet assay, we have previously demonstrated that DCQ, when combined with radiotherapy, induces DNA damage [19,22,24]. In accordance with these findings, DCQ increased the levels of the DNA damage marker gamma-H2AX in both breast cancer cell lines. Together with previous findings [24], these data provide evidence that DCQ is a potent DNA damaging agent under hypoxic conditions.

ROS are known to mediate the DNA damaging effects of hypoxia-activated prodrugs [17]. In our breast cancer system, DCQ significantly increased the levels of intracellular ROS under hypoxia after 1 hour of exposure. Interestingly, a more pronounced increase of ROS generation was observed in MDA-MB-231 (90%) than MCF-7 cells (60%), which could explain the higher sensitivity of MDA-MB-231 cells to DCQ. In fact, it has been documented that MCF-7 cells are resistant to ROS-dependent apoptosis due to the high basal levels of NRF2, a transcription factor involved in protective responses to oxidants, as well as to elevated levels of intracellular glutathione [36]. In both breast cancer cell lines, DCQ-mediated cell death appears to require ROS production since drug toxicity was reversed by the antioxidants vitamin E and DTT.

Almost 50% of breast cancer patients treated for localized disease develop metastases [25]. Evidence suggests that hypoxia contributes positively to breast tumor invasion and metastasis [25,26]. Recent studies show that hypoxia aids in the development or maintenance of breast cancer stem cells [37,38]. HIF-1α is a major molecular player by which hypoxia orchestrates such complex patterns of gene expression in most solid tumors including breast cancer [39-41]. Among other hypoxia-activated prodrugs, DCQ-induced cell death was accompanied by a reduction in the levels of HIF-1α in many cell lines [18-22]. In MDA-MB-231 cells, DCQ reduces both basal and hypoxia-induced accumulation of HIF-1α *via* proteasomal degradation. In MCF-7 cells, it has been reported that the activation of p53, by DNA damaging agents, targets HIF-1α to proteasome-dependent degradation [28]. Here we show that DCQ induces DNA damage in MCF-7 cells, however, unlike other DNA damaging agents, it does not induce a proteasome-dependent degradation of HIF-1α. Moreover, silencing p53 led to a non-significant reversal of DCQ-induced down regulation of HIF-1α. These data provide evidence that DCQ does not target HIF-1α to degradation. Since, treating MCF-7 cells with DCQ did not affect the mRNA transcript levels of HIF-1α (data not shown), we suspected a possible anti-translational activity of DCQ. One of the major oncogenic pathways that shift the balance towards HIF-1α accumulation, *via* increased translation, is the AKT/mTOR pathway [8,42-44]. In fact, Zhan et al. reported that in Hela cells, TPZ suppresses the translation of HIF-1α in an eIF2α dependent manner [29]. This suppression was accompanied by an increase in the levels of p-AKT (Ser 473) and a reduction of p-mTOR (Ser 2448) [29]. Similar to TPZ, DCQ induced an increase in the levels of p-AKT (Ser 473) and a reduction of p-mTOR (Ser 2448), suggesting a possible anti-translational activity in MCF-7 cells. Taken together, we show that DCQ reduces HIF-1α in MDA-MB-231 and MCF-7 cells *via* distinct mechanisms and possibly independently of its DNA damaging effect (Additional file 1: Figure S5).

HIF-1α is known to activate vital pathways involved in metastasis [8]. In our breast cancer system, DCQ not only reduced HIF-1α but also attenuated hypoxia-induced invasion. The reversal of hypoxia induced VEGF secretion is in accordance with the anti-angiogenic effect of DCQ documented earlier in mouse mammary epithelial cells in C57BL mice [23]. HIF-1α plays a central role in metastasis *via* direct regulation of several EMT regulators such as Snail, SLUG, and TWIST [11-14]. Here we report that targeting breast cancer cells with the hypoxia-activated drug, DCQ, reduces hypoxia-induced EMT, as indicated by the reduction of hypoxia-induced cell migration in MCF-7 cells and downregulation of TWIST in MDA-MB-231. In MDA-MB-231, the incomplete abrogation of HIF-1α and TWIST possibly explains the limited effect of DCQ on hypoxia-induced migration in this cell line [12]. In fact, exposure of MDA-MB-231 to hypoxia did not significantly alter their behavior, which may be due to the considerably high basal levels of HIF-1α in this cell line or to the constitutive activation of several oncogenic pathways involved in angiogenesis and EMT in these cells [12,13,25,45].

The present study shows that the hypoxia-activated drug, DCQ, is more effective than the clinically used drug tirapazamine (TPZ) against breast cancer cell lines. DCQ induces ROS-dependent, p53-independent apoptosis in breast cancer cell lines *in vitro*. Additionally, DCQ reduces primary tumor volume, reduces secondary site invasion, and significantly increases survival *in vivo*. DCQ exhibits its unique pro-apoptotic and anti-metastatic properties *via* the reduction of HIF-1α and downstream signaling cascades.

## Conclusions

Despite the involvement of hypoxia in breast cancer progression, no hypoxia-activated drug has been shown to be effective against breast tumors so far. Here, we show that targeting breast cancers with the hypoxia-activated drug, DCQ, induces ROS-dependent apoptosis *in vitro*, inhibits primary tumor volume, reduces secondary site invasion, and significantly increases survival *in vivo*. DCQ exhibits its unique pro-apoptotic and anti-metastatic properties *via* the reduction of HIF-1α and downstream signaling cascades. Additionally, DCQ is more effective than the clinically used drug tirapazamine (TPZ) against breast cancer, making it a viable candidate for further clinical investigations.

## Methods

### Reagents

DCQ was synthesized from 5,6-dichlorobenzofurazan oxide and dibenzoylmethane *via* the Beirut Reaction [46]. RPMI 1640, 10X trypsin-EDTA, 0.25X trypsin-EDTA, Dulbecco’s Phosphate Buffered Saline (PBS), Fetal Bovine Serum (FBS), sodium bicarbonate, penicillin-streptomycin (P/S), DMSO, trypan blue, vitamin E, and Dithiothreitol (DTT) were purchased from Sigma, St. Louis, MO, USA. Propidium iodide (PI) and CM-H_2_DCFDA were purchased from Invitrogen Molecular Probes, Eugene, OR, USA. Protease Inhibitor MG-132 was purchased from Roche Applied Science, Penzberg, Germany.

### Cell lines and cell culture

The human breast cell lines MCF-7 (p53 wildtype, noninvasive [27]), MDA-MB-231 (p53 mutant, invasive, [27]) and MCF-10A (p53 wildtype, immortalized normal breast [27]) were originally obtained from ATCC and were constantly tested for mycoplasma contamination. No authentication was performed by the authors. Cells were grown in RPMI 1640 modified medium supplemented with 10% heat-inactivated FBS and 1% Pen-Strep. All cells were maintained in a humidified atmosphere of 5% CO_2_ and 95% air. DCQ (7.5 mg) was dissolved in 1 ml of DMSO then diluted in media (DMSO ≤ 0.1%). For hypoxia exposure, cells were placed in a tightly sealed chamber (Bactron III, SHEL LAB, UK) at 37°C. The desired oxygen level was monitored by an Ohmeda Oxymeter and maintained by pumping a gas mixture of 1% O_2_, 5% CO_2_, and 94% N_2_, unless otherwise mentioned.

### Viability and clonogenic survival

Cells (10^4^) were treated with drugs 24 hours after plating. Cell viability was tested 6 hours or 24 hours after treatment by the non-radioactive cell proliferation kit (Promega Corporation, Madison, USA), an MTT-based method. Clonogenic survival was performed as described previously [19]. Briefly, cells were treated with DCQ for 3 hours under normoxia (21% O_2_) or hypoxia (1%, 10% O_2_), trypsinized, then re-plated at a density of 300 living cells/100 mm petri dish without the drug. 12 days later, cells were stained with aqueous 0.5% solution of crystal violet and the number of colonies having more than 50 cells was recorded.

### Flow cytometric analysis

Flow cytometric analysis was performed as previously described [24]. Briefly, cells were treated with DCQ at 50% confluency, and incubated for 6 hours under normoxia or hypoxia and the PI-stained DNA content was measured using Fluorescence Activated Cell Sorter (FACS) flow cytometer (Becton Dickinson, Research Triangle, NC). For Annexin V/PI, after treatment, cell pellets were washed with PBS and re-suspended in 100 μl Annexin-V-Fluos labeling solution (20 μl annexin reagent and 20 μl PI (50 μg/ml) in 1000 μl incubation buffer pH 7.4 (10 mM Hepes/NaOH, 140 mM NaCl, 5 mM CaCl_2_). Samples were incubated for 15 mins at room temperature and 0.5 ml incubation buffer was added. Cellular fluorescence was then measured by flow FACS.

### Western blot and VEGF measurement

Western blots were performed as described earlier [19]. Membranes were probed with the primary antibodies: p21 ((C-19)-G), p53 (DO-1), p-p53, HIF-1α (Novus Biologicals, Littleton, USA), gamma-H2AX, TWIST and GAPDH followed by horseradish peroxidase-conjugated anti-mouse, anti-rabbit, or anti-goat IgG-HRP (Santa-Cruz, California, USA). All blots have been generated from triplicate experiments after 6 hours of exposure to DCQ or hypoxia. The concentration of VEGF in the culture supernatant was measured using a quantitative sandwich enzyme immunoassay (R&D Systems Inc., Minneapolis, USA) according to the manufacturer’s instructions. VEGF concentrations were calculated by comparison with a standard curve generated using a 4-parameter logistic curve-fit and on-board software. VEGF concentrations were normalized to amount (pg) per 1 × 10^6^ living cells/10 ml supernatant.

### Detection of ROS

At 50% confluency, cells were incubated with 10 μM of CM-H2DCFDA in media (2% FBS) for 25 mins at 37ºC. After which, cells were washed with PBS and treated with 5 μM DCQ for 1 hour under hypoxia. Control and treated cells were trypsinized, centrifuged, washed with PBS, and fluorescence was immediately evaluated by FACS. For the antioxidant experiments, cells were pretreated with 1 mM vitamin E or DTT for 2 hours, washed with PBS, incubated with CM-H2DCFDA for 25 mins, and the above protocol was performed to determine the levels of ROS generated.

### Trans-well migration assay

2 × 10^4^ cells were seeded on top of reduced matrigel, in the trans-well upper chamber, in serum free media and the lower chamber was filled with 10% FBS media. Cells were either treated with 2.5 μM DCQ (5 μM of DCQ is toxic after 24 hours) or vehicle (supplemented in the upper chamber only) under normoxia and hypoxia for 24 hours. Then the lower side of the trans-well membrane was fixed in 4% formaldehyde and cells were stained with Hoechst and counted using fluorescence microscopy.

### Small interfering RNA (siRNA) transfections

siRNAs were used to knockdown p53 gene expression. siRNA p53 (p53 siRNA (h): sc-29435), control si RNA (Control siRNA-A: sc-37007) were purchased from Santa Cruz, California, USA. The siRNAs were transfected into MCF-7 cells using lipofectamine 2000 transfection reagent (Invitrogen, New York, USA) following manufacturer’s instructions. After 24 hours, cells were treated with DCQ placed under hypoxia for 6 hours, and proteins were extracted for western blot analysis.

### Xenograft mouse model and histological assessment

This study was approved by the Institutional Animal Care and Utilization Committee (IACUC) of the American University of Beirut. Eight weeks aged female NOD-SCID mice (average weight 23 g) were injected sub-dermally (*s.d.*) with 4 × 10^6^ MDA-MB-231 cells into the subcutaneous area of the neck region, in incomplete media. Treatment groups included DCQ (17 mg/Kg in 50 μL DMSO) or DMSO (50 μL) as control, which were administered intraperitoneally (*i.p.*), twice per week for 4 weeks. Toxicity studies have shown that 17 mg/Kg in 50 μL DMSO is a tolerated dose based on mice behavior and body weight. The weight of animals was determined daily, and the date of death was recorded. Survival curves were plotted using Kaplan-Meier method. Tumor growth and progression were monitored weekly by measurement of tumor size (length, width, height) with a caliper device. These data were plotted as the average tumor size of 22 mice/treatment group. For histological assessment, at the indicated time points (week 5, 7, and 9), 3 mice were anesthetized with isofurane and sacrificed by cervical dislocation. Biopsies from lungs, liver, and primary tumor sites were obtained. Tissues were sectioned at 6 μm thickness using a paraffin microtome and mounted on slides and stained by H&E or probed against HIF-1α or PECAM and were examined by light microscopy. Three tumors (week 5) per treatment group (DMSO *versus* DCQ) were assayed for lumen containing micro-vessels. Micro-vessels were counted from four random fields at low magnification.

## Abbreviations

AQ4N: di-N-oxide analogue of mitoxantrone [1,4-bis 5,8-dihydroxy-anthracene-9, 10-dione banoxantrone]; CSC: Cancer stem cells; CM-H2DCFDA: 5-(and-6)-chloromethyl-2′,7′-dichlorodihydrofluorescein diacetate, acetyl ester; DCQ: 2-benzoyl-3-phenyl-6,7-dichloroquinoxaline-1,4-dioxide; DMSO: Dimethylsulfoxide; DTT: Dithiothreitol; EMT: Epithelial to mesenchymal transition; FBS: Foetal Bovine Serum; HCR: Hypoxia cytotoxicity ratio; HIF: Hypoxia inducible factor; IC50: Half maximal inhibitory concentration; mTOR: Mammalian target of rapamycin; PBS: Phosphate Buffered Saline; ROS: Reactive oxygen species; TPZ: Tirapazamine; TUNEL: Terminal deoxynucleotidyl transferase dUTP nick end labeling; VEGF: Vascular endothelial growth factor.

## Competing interests

The authors declare that they have no competing interests.

## Authors’ contributions

KIG: Conception and design, Development and methodology, Acquisition of data, Analysis and interpretation of data, Writing and revision of the manuscript. SBES: Acquisition of data, Writing and revision of the manuscript. KAZ: Development and methodology, Acquisition of data, Analysis and interpretation of data. STR: Development of DCQ. MJH: Study supervision, Conception and design, Development of DCQ. MEES: Corresponding author, Conception and design, Development and methodology, Analysis and interpretation of data, Revision of the manuscript, Study supervision. HUGM: Corresponding author, Conception and design, Development and methodology, Analysis and interpretation of data, Writing and revision of the manuscript, Study supervision. All authors read and approved the final manuscript.

## Supplementary Material

Additional file 1: Figure S1 DCQ spares normal breast cell lines under normoxia. MTT viability assay was performed after 6 hours of exposure to DCQ under normoxia (21%). Results (Average ± SE) are from triplicate measurements from 3 independent experiments. **Figure S2.** DCQ reduced viability of breast cancer cell lines more efficiently than TPZ. Trypan blue exclusion assay was performed on MDA-MB-231 and MCF-7 cells exposed to DCQ and TPZ. 15 x 10^4^ cells were seeded in 6 well plates, 24 hours later, cells were treated with the indicated concentrations of TPZ for 6 hours under normoxia (21% O_2_) or hypoxia (1% O_2_), and were harvested 24 hours post treatment for MTT and trypan blue assays. Results are of triplicate experiments. **Figure S3.** DCQ induces apoptosis in breast cancer cell lines, preferentially under hypoxia. Cell cycle analysis was performed on cells exposed to DCQ (IC_50_) for 6 hours under normoxia or hypoxia and DNA content of PI stained cells was determined 24 hours later. The percentage of PreG1 was determined using CellQuest software and the averages ± SD were obtained from the results of at least two independent experiments each done in duplicate. **Figure S4.** DCQ reduces HIF-1α in MDA-MB-231 in a ROS-independent mechanism. MDA-MB-231 were pretreated with DTT for two hours, washed with PBS, then treated with DCQ (5 μM). Whole cell lysates of MCF-7 were prepared after 6 hours of exposure to DCQ under hypoxia, and blots were probed for HIF-1α and GAPDH. **Figure S5.** DCQ reduces HIF-1α in MDA-MB-231 and MCF-7 *via* distinct mechanisms. In MCF-7 cells, DCQ inhibits the accumulation of HIF-1α by reducing its synthesis, however, in MDA-MB-231 DCQ induces proteasomal degradation of the protein. In both cell lines DCQ enhances p-H2AX expression, and induces ROS-dependent apoptosis. Click here for file
