# Supplementary material for: The quinoxaline di-N-oxide DCQ blocks breast cancer metastasis in vitro and in vivo by targeting the hypoxia inducible factor-1 pathway
Source: Mol Cancer. 2014 Jan 24;13:12. doi: 10.1186/1476-4598-13-12 (PMC3932516; doi:10.1186/1476-4598-13-12)
Supplement: Additional file 1: Figure S1 — DCQ spares normal breast cell lines under normoxia. MTT viability assay was performed after 6 hours of exposure to DCQ under normoxia (21%). Results (Average ± SE) are from triplicate measurements from 3 independent experiments. Figure S2. DCQ reduced viability of breast cancer cell lines more efficiently than TPZ. Trypan blue exclusion assay was performed on MDA-MB-231 and MCF-7 cells exposed to DCQ and TPZ. 15 x 104 cells were seeded in 6 well plates, 24 hours later, cells were treated with the indicated concentrations of TPZ for 6 hours under normoxia (21% O2) or hypoxia (1% O2), and were harvested 24 hours post treatment for MTT and trypan blue assays. Results are of triplicate experiments. Figure S3. DCQ induces apoptosis in breast cancer cell lines, preferentially under hypoxia. Cell cycle analysis was performed on cells exposed to DCQ (IC50) for 6 hours under normoxia or hypoxia and DNA content of PI stained cells was determined 24 hours later. The percentage of PreG1 was determined using CellQuest software and the averages ± SD were obtained from the results of at least two independent experiments each done in duplicate. Figure S4. DCQ reduces HIF-1α in MDA-MB-231 in a ROS-independent mechanism. MDA-MB-231 were pretreated with DTT for two hours, washed with PBS, then treated with DCQ (5 μM). Whole cell lysates of MCF-7 were prepared after 6 hours of exposure to DCQ under hypoxia, and blots were probed for HIF-1α and GAPDH. Figure S5. DCQ reduces HIF-1α in MDA-MB-231 and MCF-7 via distinct mechanisms. In MCF-7 cells, DCQ inhibits the accumulation of HIF-1α by reducing its synthesis, however, in MDA-MB-231 DCQ induces proteasomal degradation of the protein. In both cell lines DCQ enhances p-H2AX expression, and induces ROS-dependent apoptosis. [file 1476-4598-13-12-S1.ppt]

## Slide 1
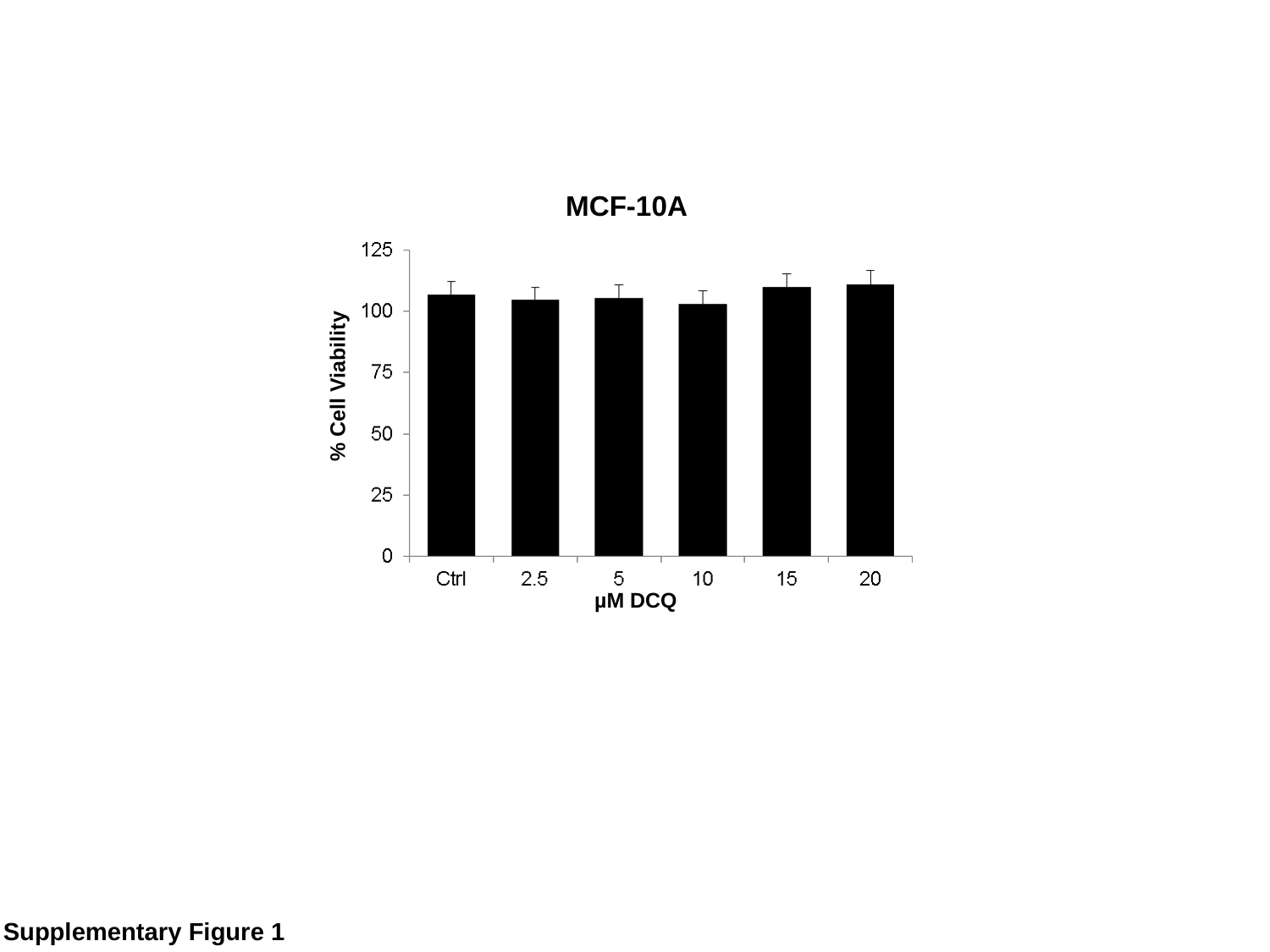

MCF-10A
% Cell Viability
µM DCQ
Supplementary Figure 1

## Slide 2
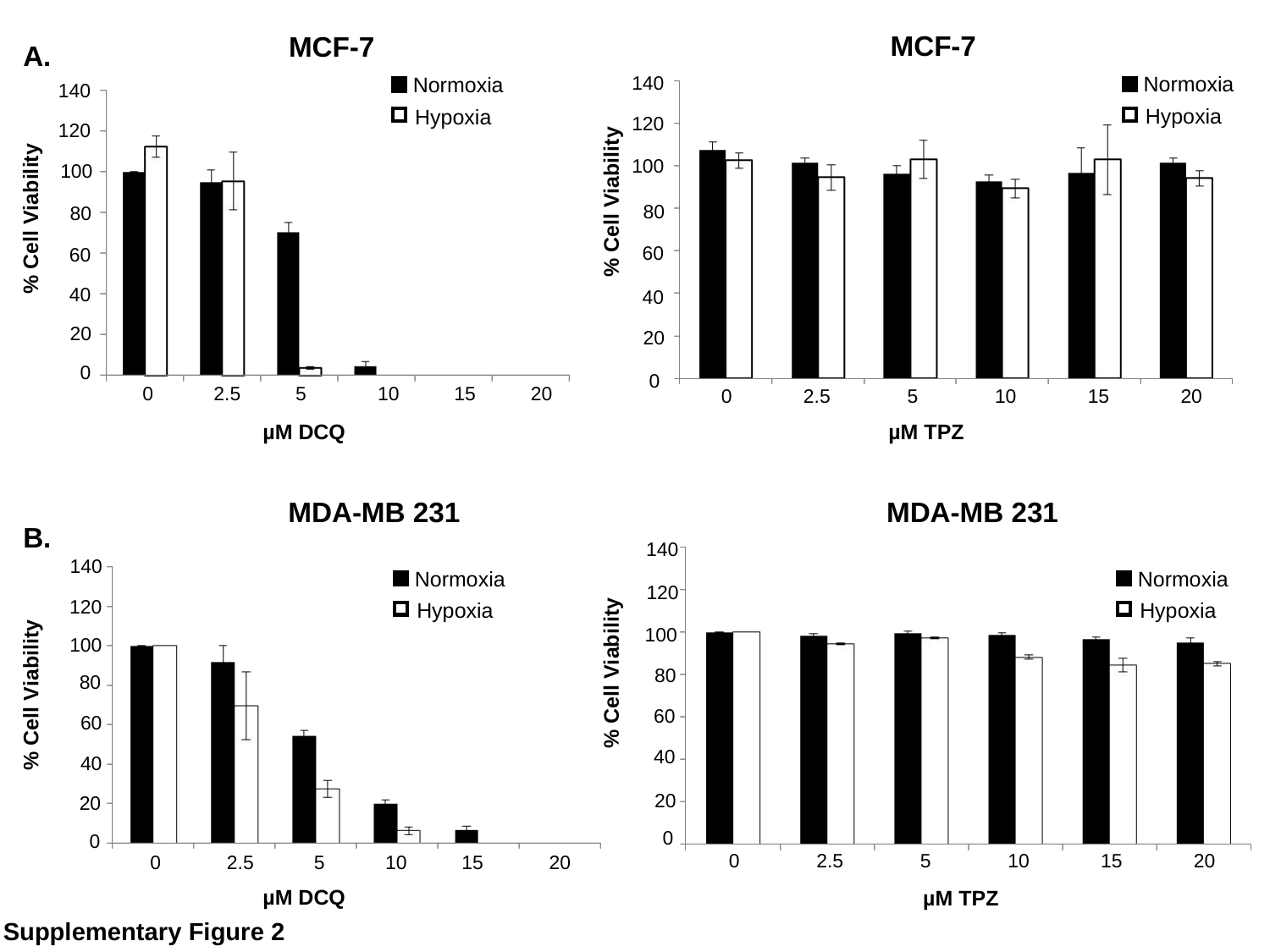

MCF-7
140
120
100
80
60
40
20
0
% Cell Viability
0 2.5 5 10 15 20
µM DCQ
MCF-7
140
120
% Cell Viability
100
80
60
40
20
0
0 2.5 5 10 15 20
µM TPZ
A.
Normoxia
Hypoxia
Normoxia
Hypoxia
MDA-MB 231
0 2.5 5 10 15 20
µM TPZ
140
120
% Cell Viability
100
80
60
40
20
0
MDA-MB 231
120
% Cell Viability
100
80
60
40
20
0
0 2.5 5 10 15 20
µM DCQ
B.
140
Normoxia
Hypoxia
Normoxia
Hypoxia
Supplementary Figure 2

## Slide 3
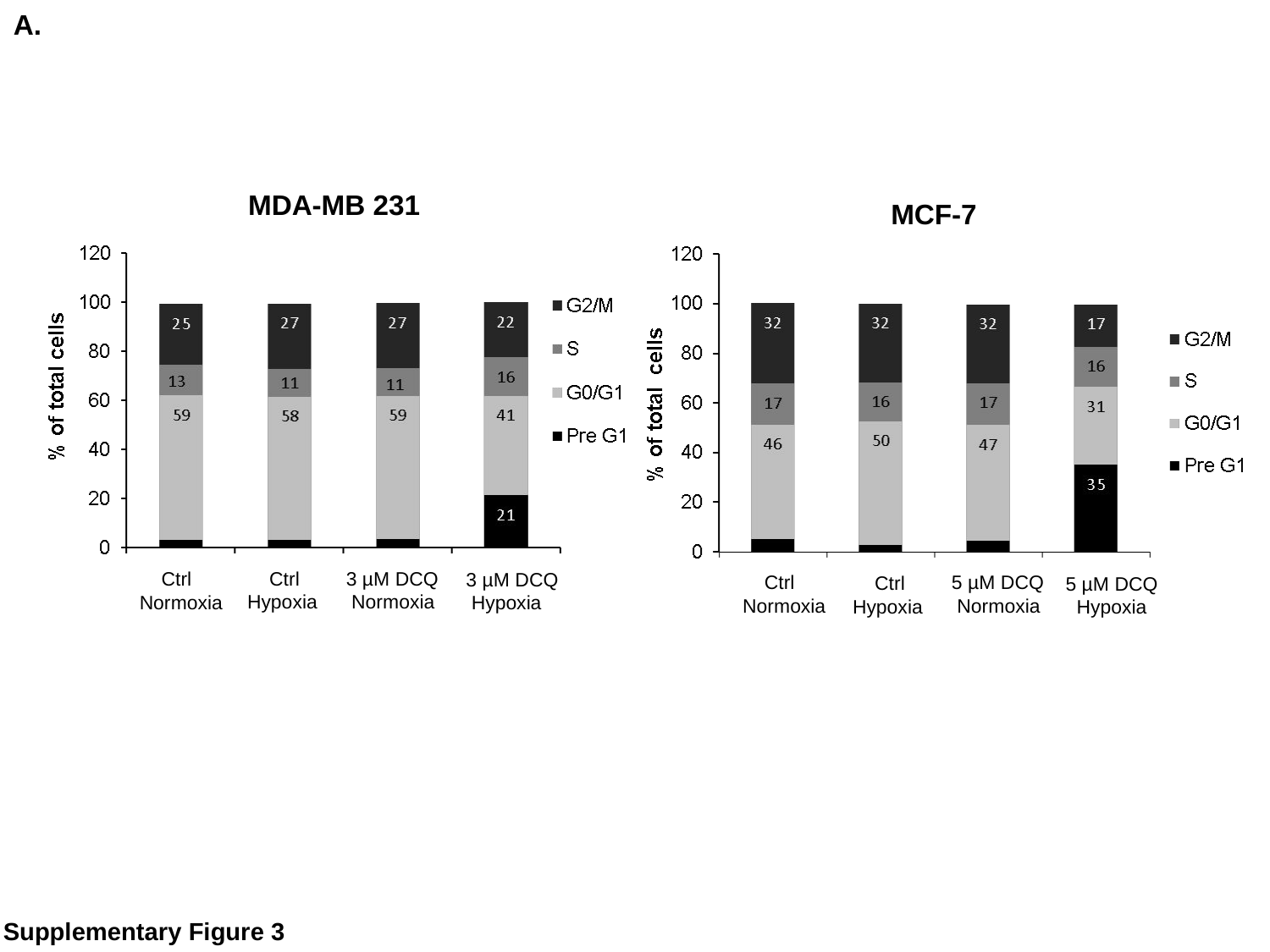

A.
MDA-MB 231
MCF-7
 Ctrl
Normoxia
5 µM DCQ
 Normoxia
 Ctrl
Hypoxia
5 µM DCQ
 Hypoxia
 Ctrl
Hypoxia
 3 µM DCQ
 Normoxia
 Ctrl
Normoxia
 3 µM DCQ
 Hypoxia
Supplementary Figure 3

## Slide 4
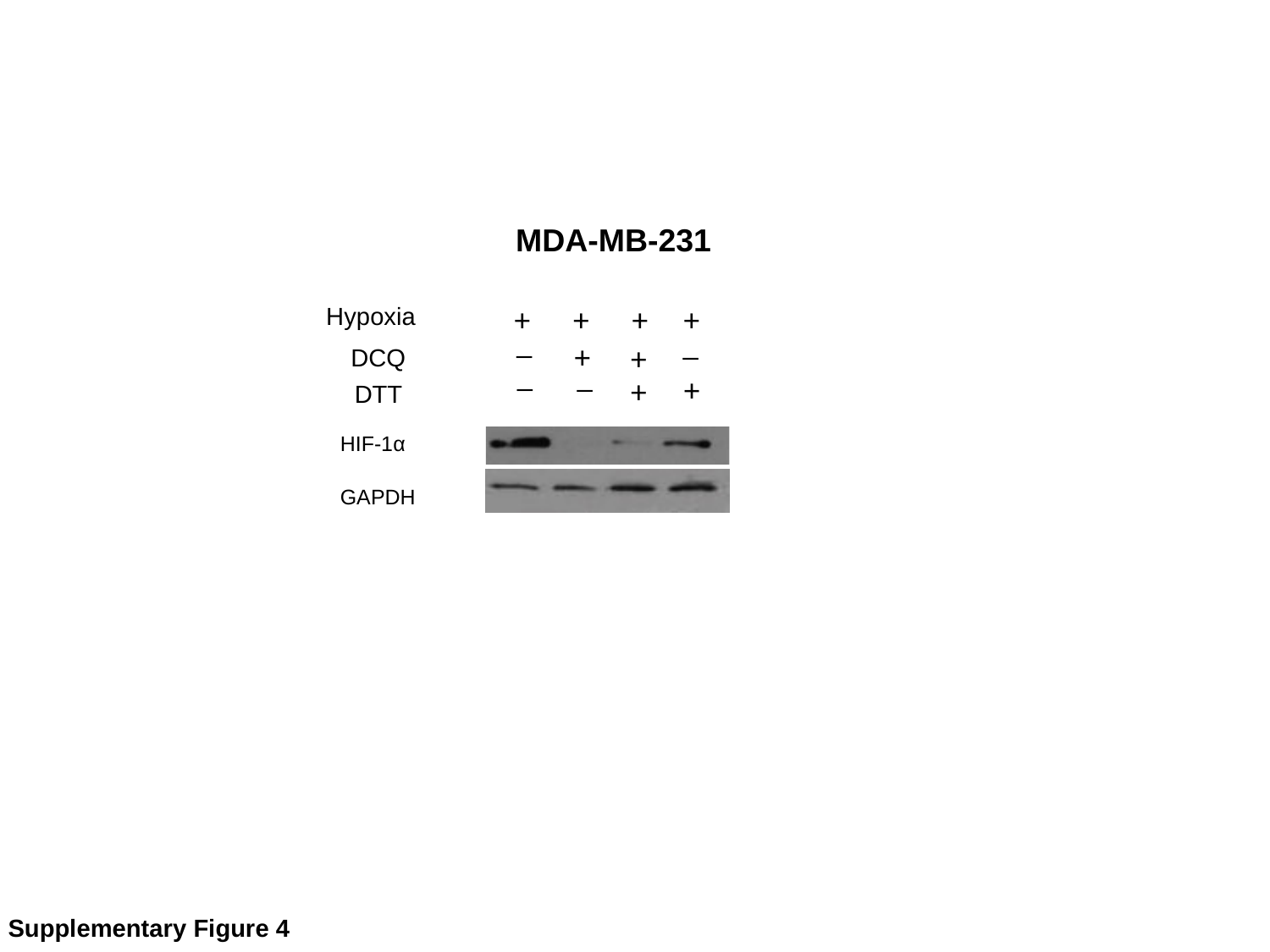

MDA-MB-231
+ + + +
Hypoxia
_
_
+
+
DCQ
_
_
+
+
DTT
HIF-1α
GAPDH
Supplementary Figure 4

## Slide 5
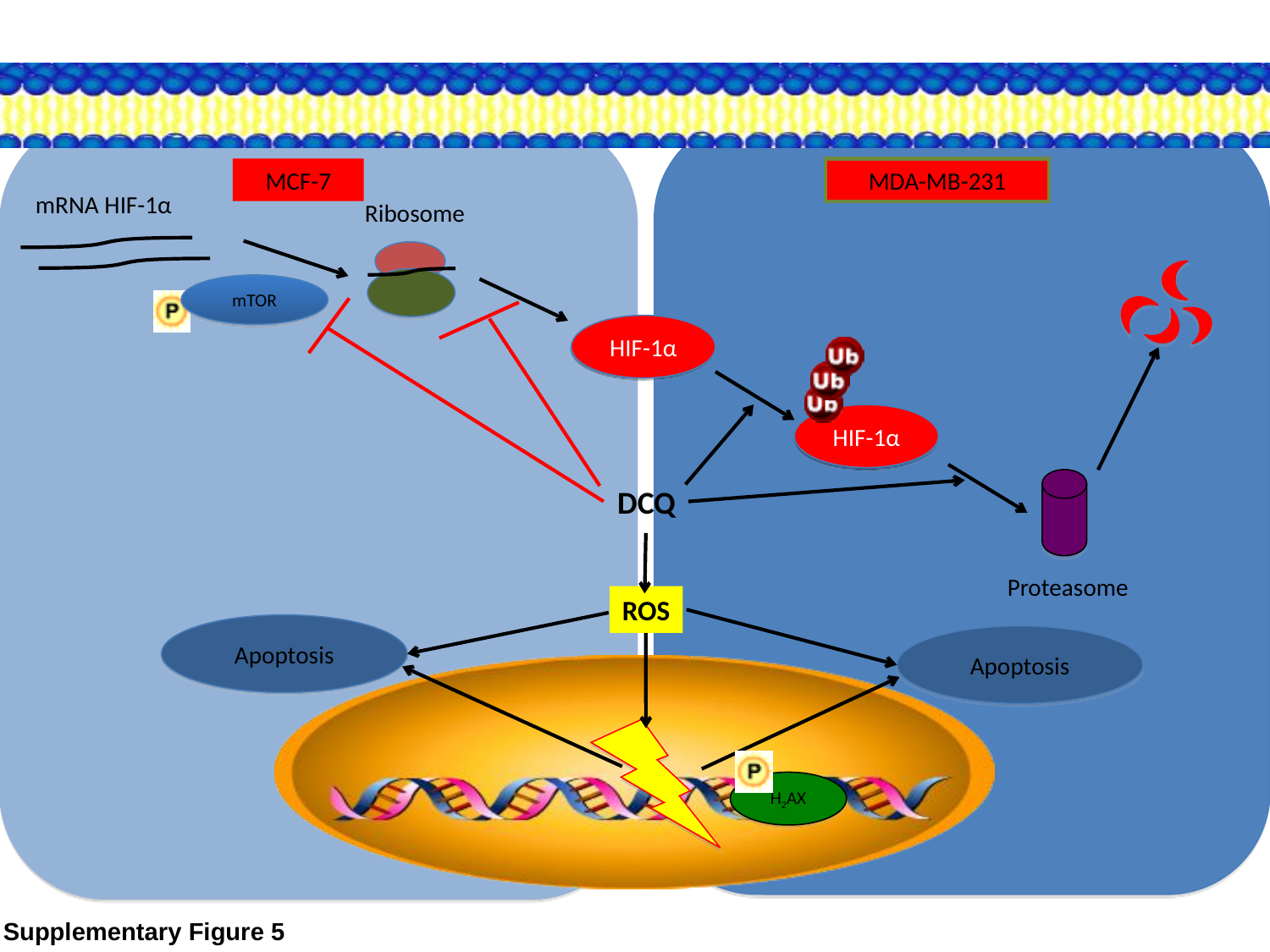

MCF-7
MDA-MB-231
mRNA HIF-1α
Ribosome
mTOR
HIF-1α
HIF-1α
DCQ
Proteasome
ROS
Apoptosis
Apoptosis
H2AX
Supplementary Figure 5
